# Microwave Moisture Sensing of Seedcotton: Part 1: Seedcotton Microwave Material Properties

**DOI:** 10.3390/s16111843

**Published:** 2016-11-02

**Authors:** Mathew G. Pelletier, John D. Wanjura, Greg A. Holt

**Affiliations:** United States Department of Agriculture, Agricultural Research Services; Cotton Production & Processing Research Unit, Lubbock, TX 79403, USA; John.Wanjura@ars.usda.gov (J.D.W.); Greg.Holt@ars.usda.gov (G.A.H.)

**Keywords:** microwave, moisture, cotton, seedcotton, seed, density independent

## Abstract

Moisture content at harvest is a key parameter that impacts quality and how well the cotton crop can be stored without degrading before processing. It is also a key parameter of interest for harvest time field trials as it can directly influence the quality of the harvested crop as well as skew the results of in-field yield and quality assessments. Microwave sensing of moisture has several unique advantages over lower frequency sensing approaches. The first is that microwaves are insensitive to variations in conductivity, due to presence of salts or minerals. The second advantage is that microwaves can peer deep inside large bulk packaging to assess the internal moisture content without performing a destructive tear down of the package. To help facilitate the development of a microwave moisture sensor for seedcotton; research was performed to determine the basic microwave properties of seedcotton. The research was performed on 110 kg micro-modules, which are of direct interest to research teams for use in ongoing field-based research projects. It should also prove useful for the enhancement of existing and future yield monitor designs. Experimental data was gathered on the basic relations between microwave material properties and seedcotton over the range from 1.0 GHz to 2.5 GHz and is reported on herein. This research is part one of a two-part series that reports on the fundamental microwave properties of seedcotton as moisture and density vary naturally during the course of typical harvesting operations; part two will utilize this data to formulate a prediction algorithm to form the basis for a prototype microwave moisture sensor.

## 1. Introduction

Moisture content, MC, at harvest is a key parameter that impacts quality and how well the agricultural crops can be stored without degrading before processing. It is also a key parameter of interest for harvest-time field trials as it can directly influence the quality of the harvested crop as well as alter the results of in-field yield and quality assessments. Microwave sensing of moisture has several unique advantages over lower frequency sensing approaches. The first is that microwaves are insensitive to variations in conductivity, due to presence of salts or minerals in the dust and dirt on the crop. The second advantage is that microwaves can peer deep inside large bulk packaging to assess the internal moisture content without performing a destructive teardown of the package. Finally, microwave sensing has been shown to provide a high-accuracy rapid means to assess bulk moisture of materials. In order to develop a prototype microwave sensor, knowledge of the basic microwave material properties is required and, to date, there are no references that the authors are aware of reporting on the microwave properties of seedcotton that could be utilized to help develop a microwave moisture sensor for seedcotton modules. The only other pertinent references relating microwave sensing of moisture to cotton are the earlier works by the authors in sensing of cotton lint moisture as packaged in high-density cotton bales [1,2,3,4]. Of importance is that this work is only partially applicable as cotton bales are pressed to a much higher density. Moreover, cotton lint is very different from seedcotton as it is comprised of multiple constituents; hence the response is not expected to be the same. Further, those publications predominantly discuss measurement techniques and do not include microwave material response with respect to moisture and density. The only other reference that would loosely apply, as it addresses fibrous materials, is the work performed at 10+ GHz using a gunn oscillator [5]. The primary reason this work is not more pertinent to this research effort is that it is the response of seedcotton when subjected to free-space through-transmission sensing of large bulk packaging (0.5 m to 2 m thick) that is of particular interest here. Additionally, preliminary work by the authors suggests that, in order to avoid extremely high attenuation of signals, the likely optimum range will be below 2.5 GHz range; above this frequency, the attenuation for wet modules will be too great to provide reliable measurements. Given this targeted lower microwave frequency range, of importance is the dearth of reported literature on prediction equations that work in the low GHz region for cotton fiber and seedcotton, although there may be some useful techniques or equations that could be transferred from the higher microwave region to the lower frequencies of interest herein. A section later in this paper will cover the techniques and algorithms that have been successfully utilized for measurement of moisture using these high frequency microwave regions. The overall objective of this research is to help facilitate the development and design of a moisture sensor for bulk-packaged seedcotton micro-modules (110 kg) and commercial scale modules (13.5 metric tons). To achieve the overall objective, this study focused on the following:

(1) Presenting the basic microwave material properties of seedcotton (part one);
(2) Developing prediction equations relating microwave material properties to moisture content of bulk-packaged seedcotton, given there is a known weight of the bulk package (part two);
(3) Assessing accuracy of previously reported 9–15 GHz density-independent equations (developed for use in corn and wheat) for prediction of moisture in seedcotton in the 1–2.5 GHz band, without the need for weighing the material (part two);
(4) Examine the potential of density-independent prediction equations relating microwave material properties to moisture content of bulk-packaged seedcotton (part two).

To help facilitate the experimental designs for the collection of microwave properties of seedcotton, a literature review of the art of microwave moisture sensing was performed and the key pertinent elements will be presented in the next section.

### 1.1. Literature Review: Theory

In developing a moisture sensor for seedcotton, it is instructive to examine each of the constituents at a molecular and physical layer. Seedcotton is a composite material comprised of cotton lint, seeds and woody by-products such as sticks and cotton flower bracts, “cotton burs” and a significant amount of air. All of the physical components are mainly comprised of some form of cellulose. The exception to this is the seed which has a significant amount of oil: 30%–40% by seed-weight. In a typical harvested batch of seedcotton, the constituent partitioning varies depending upon the type of harvester utilized. This research is targeted for use on stripper harvesters that typically bring in a partitioning of 30% seeds, 30% cotton lint and the rest that is comprised of; sticks, burs and leaf trash. Of note is that cotton is typically dried and defoliated so that the leaves are normally dry and have very low water content at or below 15% and are thereby a minor fraction by weight. From this fractionation, it is apparent that the primary constituents are some form of cellulose and a small fraction of seed oil. The primary difference between wood cellulose and cotton lint cellulose is the degree of crystallinity in the polymerization. In the crystalline areas of the cotton lint fibers, the tightly packed fibers are typically bound to their neighboring fibers through hydrogen bonding on the hydroxyl groups associated with each cellobiose repeat unit. As such crystalline cellulose typically holds less water than amorphous cellulose, which has a much higher degree of available hydroxyl units that are free to chemisorb water.

**Figure 1:**
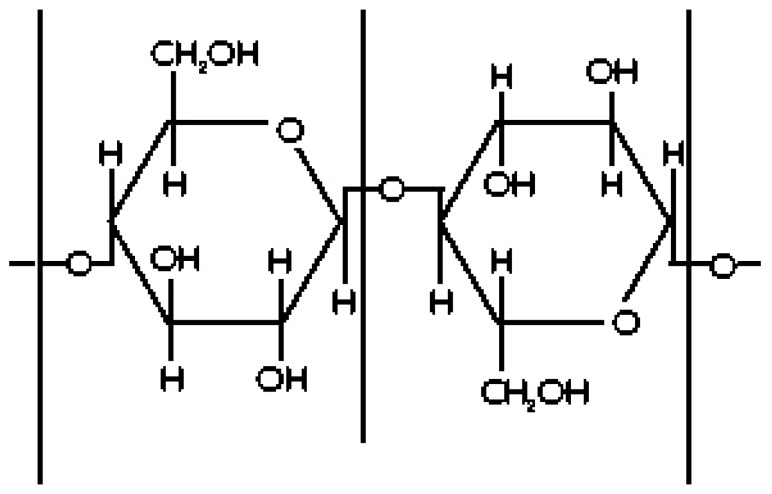
Drawing illustrates the chemical structure of cellulose detailing the hydroxyl, “OH”, water sorption sites.

Cellulose is a macromolecule––a polymer made up of a long chain of glucose molecules linked together as beta-cellobiose repeat units (Figure 1). In each unit there are three hydroxyl groups, one primary and two secondary, that are chemically reactive. These hydroxyl groups serve as the principal sorption sites for water molecules. Water that is directly sorbed is firmly chemisorbed onto the cellulosic hydroxyl groups by hydrogen bonding.

The physical structure of cotton fibers creates additional means to adsorb water. The cotton fiber is comprised of varying-sized capillary pores and networks. This structure enables a significant amount of water to be held by the fiber beyond the fraction of which is chemisorbed directly to the available hydroxyl units on the fiber molecule. Of importance to electrical sensing as the cotton fiber becomes wetter is that more of the water held by the cotton fiber is closer to the form of free water than that of chemisorbed bound water. This is especially true as cotton lint approaches saturation, which occurs from 20%–24% moisture content, wet basis. For normally harvested seedcotton, the cotton lint fraction of the water is predominantly in the bound form, ranging from one to a few mono-layers of water. As a field- or harvester-based sensor should be able to alert the operators of abnormally wet modules, the sensor should have an extended range.

**Figure 2:**
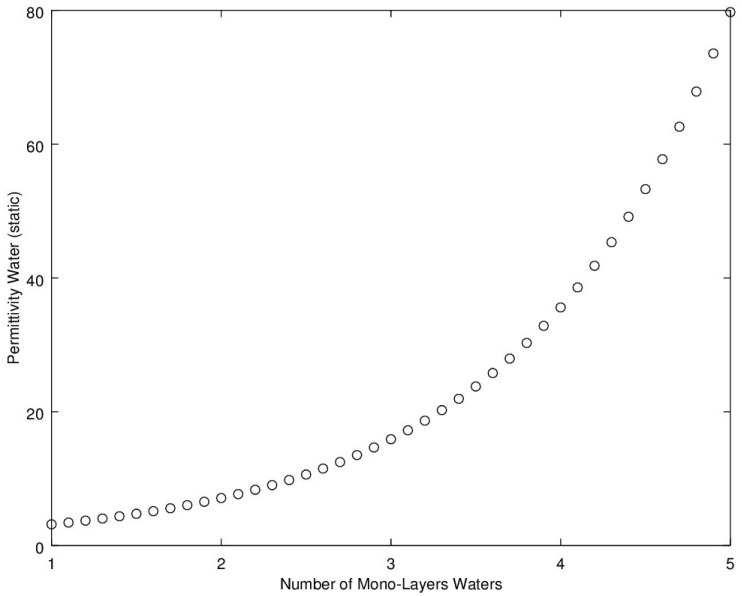
Detail illustrating how the real portion of complex permittivity of bound water, at 20 °C, varies as the water molecules move away from the surface of the material. Mono-layer 1 is closest to the surface of the material (each mono-layer of water is approximately 1.5 × 10^−10^ m), and exhibits a permittivity nearly equivalent to that of ice (water in a tightly bound crystalline matrix form that is highly resistant to pivoting in a rapidly rotating electric-field).

Of interest to the development of a moisture sensor is how a water molecule behaves in a rapidly rotating electric field, when they are on and around the fibers, either in bound form or as free water in the intra-fibril spaces inside each cotton lint fiber. In addition to the internally held water, when cotton approaches saturation, free water can also build up on the surface of the fibers. When water is attached to the hydroxyl binding sites, it becomes fixed in space and, being a polarized molecule, it attracts an additional water molecule to attach to it via weak electro-statically charged hydrogen bonding. As each layer of water builds up on the lower level layers, the number of hydrogen bonds between each water molecule increases, and with each additional bond sharing, the total attraction force becomes lower. As the attraction force decreases, each molecule is held progressively looser, thereby allowing each additional layer of water to more closely follow a rapidly changing electric field. Water molecules in free water, at 20 °C, are bounded to other water molecules through an average of 3.5 hydrogen bonds which represents the normal upper limit. However, these varying levels of constraints on the water molecules as the water moves away from the surface of the polymer leads to a variation in the ability of the electric field to store energy. The measure of this energy storage can be quantified as the dielectric-constant, otherwise known as the relative electrical permittivity. In essence, the energy is stored in the hydrogen bonds of the water molecules and between the water molecules and the hydroxyl binding sites. Figure 2 depicts a model of how the dielectric-constant of water varies as it moves away from the surface of the molecule. The model shown was proposed for use in soils [6,7,8,9]. As this phenomenon is based primarily upon water to water hydrogen binding, it is anticipated that this trend should be attributable for most materials with the curve shape, albeit varying due to specific surface area of the material and the attraction force of the material to water molecules.

The next important physiological trait that influences the relation between electrical permittivity to MC is the specific surface area of the material. This influence is an extension of the effect on permittivity by the water molecule’s distance from the surface. This can be explained as with high surface area materials; for the same moisture content, the amount of water in the lower mono-layers is greatly increased. Hence, for the high surface area materials such as clay, cellulose and lignin, they all produce a depressed permittivity in relation to lower surface area materials such as silt and sand [6,10,11,12]. The electrical response for bound water in biological constituents is more nebulous; the specific surface is dependent upon the water content. It is reported that the trends, readily measured for free water, hold for bound water with several models having been proposed and reported [10,12,13]. Of interest is that for bound water, many materials have variable specific surface areas as they tend to unfold their physical structures in response to wetting. This feature makes the assessment of the response of bound water’s response to electric fields a moving target as it is hard to predict exactly which mono-layer level the water molecules are attached to.

**Figure 3:**
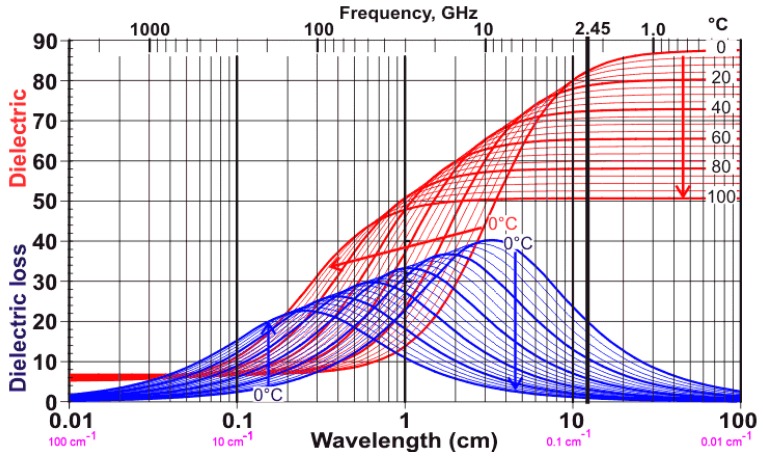
Complex permittivity of water as a function of frequency (Reproduced with permission [17]).

The final influencer on permittivity is temperature. The trend almost universally follows that of free water [14,15] where the dielectric constant and loss in agricultural materials and soils both become depressed as the temperature increases [10,15,16]. This trend for free water is illustrated in Figure 3 where it can be seen that, as the temperature increases, the dielectric constant (also known as real component of complex permittivity; which is the energy storage component), and the dielectric damping (loss term; also known as the imaginary component of complex permittivity) are attenuated.

As discussed in the earlier paragraphs, for prediction of moisture content with microwaves, water is the primary component in most agricultural materials that stores energy and can be measured by assessing the material’s electrical permittivity. Conversely, the dry bulk material, without water, cannot readily align itself with a changing electric field at microwave frequencies as the molecules are encased in a crystal-like polymer matrix and hence exhibit a very low dielectric constant that is similar or lower than that of ice, [15]. Looking to the literature for insight into how the bulk dry solids affect the signal, von Hippel, 1995 reported on the microwave permittivity of dry paper, which is primarily cellulose, hemi-cellulose and lignin. The results suggest that the dry cellulose should provide a bulk dielectric constant, ε’, below that of ice (ε’ = 2.75) and one that is largely independent of frequency below 3.0 GHz with a dielectric loss, ε’’, of 238 at 300 MHz and 203 at 3.0 GHz. Of note is that both cotton lint and seedcotton have much lower bulk densities, so these values are expected to be significantly reduced accordingly.

For seeds, the other main constituent of seedcotton, reference [18] reported dielectric constants, for 1.0 GHz, ranging from 2.3 (at 6% MC) to 2.7 (at 12% MC) and loss terms ranging from 0.15 (at 6% MC) to 0.28 (at 12% MC). One further delineation between cottonseeds and cereal crop seeds such as wheat and corn lies in the much higher oil content for cottonseeds (30%–40%).

Literature-reported values for oil are stable across a wide frequency range and typically low, with dielectric constants ranging from 2 to 4 [15]. Given the frequency stability of dielectric constant of the seeds, a multi-frequency approach may be able to separate the response of oil from that of bound water.

Concern has been expressed regarding the potential influence from salts into the microwave measurements. In examining this issue in soil research, [19] compared time-domain reflectometry, TDR, and microwave response using a network analyzer and a high-quality through-transmission line where it was determined that salt concentration did not affect microwave measurements above 500 MHz. Given these results, coupled with the target frequency range being well above 500 MHz, this study will not examine the influence of any potential variation due to any salt contamination that might occur from settling of various types of soils through dust accumulation on the cotton bolls.

In summary, the following traits are expected from the microwave response:

(1) Seedcotton is a complex material comprised of five main constituents (air, water, burs, sticks, seeds and cotton lint). As such, stripper harvested cotton, with a high by-product content of burs and sticks (30% by mass), is expected to exhibit a different microwave response than cleaner seedcotton such as that harvested with picker harvesters (6% by-product content).
(2) The volumetric air content of seedcotton is significantly larger than the materials reported in the literature (density of seedcotton varies from 0.28–0.36 g·cm^−3^ whereas wheat and corn are nearly twice this value). Hence, it is expected that seedcotton will exhibit a measurable depression in the electrical permittivity of the bulk seedcotton, in comparison to cereal crops’ response.
(3) Due to the character of free water’s microwave response, as a function of frequency, 10+ GHz X-band, and above, responses are likely to be very different than those observed from the lower 1–2 GHz region of interest to this research (Figure 3).
(4) Temperature depresses the permittivity, and dielectric loss of free water and water in bulk matrix of materials have been widely reported to follow this same trend.
(5) High specific surface area materials have a reduced permittivity response, in comparison to low specific materials. Hence it is expected that equations may be reproducible between materials with similar specific surface areas. Conversely, the microwave response is very likely to differ as the surface area of the material of interest moves away from the model material.
(6) Minimal to no measurable response to salts from particulate accumulation of dust due to wind transport of erodible top-soils.

### 1.2. Literature Review: Measurements

One measurement method suitable for sensing moisture of large bulk packaged materials is interrogation of electrical permittivity. The most suitable technique for these types of packages is to transmit microwave energy through the material and measure the response on the far side of the material via a through-transmission measurement [2,3,4,20,21,22,23]. The measurement typically consists of quantifying the signal loss and time delay of the signal after it has passed through the material. Equation (1) shows the relation between measured propagation constant and the complex permittivity, as derived from first principles using Maxwell’s equations for electromagnetic propagation of plane waves in a non-magnetic media, [24].

γ = α + jβ = jω[μ_ο_ε’(1 − j(ε”/ε’))]^0.5^ (1)

where

γ ≡ complex propagation constant
α ≡ propagation attenuation constant (neps·m^−1^)
β ≡ propagation delay constant (rads·m^−1^)
ε’ ≡ real portion of complex electrical permittivity (dielectric constant) (F·m^−1^)
ε” ≡ imaginary portion of complex electrical permittivity (dielectric damping)
j ≡ imaginary number = [−1] ^0.5^
ω ≡ frequency of electromagnetic wave (rads·s^−1^)
μ_ο_ ≡ permeability of free space (H·m^−1^)

For convenience we note the typical usage equation, which is based on the solution of the Helmholz wave equation for the electric field, “E”, propagating in the z-direction in a source-less, non-magnetic environment as Equation (2).

E_x_(z,t) = e^−γz^ = e^−(α + jβ)z^ = e^−αz^ cos(ωt + βz) (2)

As Equation (1) is not easily inverted to isolate ε’ or ε”, in practice, it is useful to utilize a power series approximation to provide a first-order model that is valid for low-loss materials. While this approximation is widely reported, the derivation is not and was therefore developed and reported in [4], as it provides insight into when this approximation is valid and the costs associated therewith. By definition, a low loss material is where ε”/ε’ << 1.0. Figure 3 provides the data required to estimate the loss ratio, as a function of temperature, across the frequency range of interest. For use in seedcotton sensing of bulk packaged micro and commercial modules, our preliminary tests indicate the most suitable range is from 1.0 to 2.5 GHz. For this range, the error for using the first-order model is less than 0.22% for ε” and less than 0.55% for ε’. The simplified first-order model is detailed in Equations (3) and (4).

β = ω[μ_ο_ε’]^0.5^ = (ω/c)[ε_r_’]^0.5^ (3)

α = 0.5β[(ε_r_”/ε_r_’)] (4)

where c ≡ speed of light (m·s^−1^)

In review of the literature on microwave transmission measurements for moisture sensing [25,26] established an X-band (10 GHz) system for fish-meal, wheat, coffee and milk powder. Their prediction equation revolved around the ratio R = A/θ where “A” is attenuation (dB), and “θ” is phase delay (rads). In regressions of R versus θ, they reported that R was useful as a linear predictor of MC, for a known density. They reported that the R metric was slightly less sensitive to density variations than ε_r_”/(ε_r_’ − 1). Reports in [21,22], noted a curvilinear relation between R to MC, for a mono-frequency measurement also in X-band. Another approach [27], proposed using the attenuation ratio of (A_12 GHz_)/(A_9 GHz_) for use in timber and green tea and [28] reported on a 9 GHz variation for R where they found MC = 109.9[R]^0.5^. On a continuation of that work, further research along this vein saw the development of an alternative density-independent function mc = [ε”/(ε’(a*ε’ − ε”))]^0.5^ that was reported to work at 11.3, 14 and 18 GHz when used across the density range of 0.72–0.85 g·cm^−3^ (wheat) and 0.69–0.83 g·cm^−3^ (corn) [29].

In summary, the main terms related to density-independent moisture content predictions from the literature are noted in Equations (5)–(8) [21,25,26,28,29].

mc = aR (5)

mc = aR + b(A)(6)

mc = a(R)^0.5^ (7)

mc = [ε”/(ε’(a*ε’ − ε”))]^0.5^ (8)

where

a and b ≡ proportionality constants
mc ≡ moisture content
θ ≡ phase delay of signal caused by insertion of material (rads)
A ≡ attenuation of signal (# from 0:1)
R ≡ (A/θ)

Given the high performance as reported in the literature for Equations (5)–(8) (at high microwave frequencies), this research will assess these equations for their ability to predict MC for seedcotton at the frequency range of interest to this research (1–2.5 GHz). Due to the expected difficulty in translating the high performance equations from the elevated frequency ranges (9–18 GHz), a full report of microwave properties as they relate to moisture content, density and dry bulk density will be explored and reported for later use in part two’s equation and sensor development report.

## 2. Materials and Methods

This research was conducted to measure and report on the pertinent microwave properties, to enable sensor development for use on typical stripper-harvested seedcotton. This information is reported in a manner that will provide the basic information to enable a sensor development company the required specifications needed to design a working prototype microwave moisture sensor, or imager, for use on both 110 kg micro and standard 10 mt cotton modules.

Freshly harvested seedcotton is a non-homogeneous material comprised of several primary constituents (water, cottonseeds, cotton lint, cotton flower bracts (burs), sticks and dried leaves). To ensure test results conform as closely as possible to the target deployment, and as the water is mobile between fractions of moisture on the surface to the adsorbed water fraction; ideally the microwave characterization measurements would be performed as close to harvest time as possible. As such, the main factors of interest to sensor development are the moisture content and the density (as an increase in density adds more water molecules than the microwave signal has to propagate through). To assess seedcotton microwave properties, a commercial variety of seedcotton (Dowagro’s Phytogen 333), grown under typical commercial conditions, was harvested with a field-cleaner equipped stripper-style cotton harvester. Of importance is that stripper harvesters typically bring in 2–3 times as much by-product than picker-style harvesters do. The main constituents, by mass, of the by-products are sticks and wood cotton flower bracts (burs).

Crop preparation followed standard commercial practices of pre-harvest defoliation followed by an extra-step of desiccating the crop with paraquot, standard practice for stripper harvesting. Immediately following the harvesting operation, the harvested seedcotton was immediately packed into 100 kg micro-modules, weighed and then, within one hour, scanned on a microwave network analyzer to allow for extraction of the microwave properties of the seedcotton. To encompass the full range of microwave responses, the modules for the test were designed to incorporate a wide span of moisture and densities that are typical for normal harvest operations. The objective of the selected density range was to encompass the typical span pertinent to cotton harvested and packaged into modules in the West Texas growing region that utilize stripper harvesters. The microwave measurements taken on the micro-modules were designed to allow for extraction of microwave material properties. The measurements provide the pertinent information for developing sensors based on microwave free-space through-transmission measurements specific to seedcotton that is stripper harvested (which includes a high amount of extraneous matter, such as sticks and woody cotton flower bracts, “burs”).

### Experimental Design

The objective of the experimental portion of this study is to obtain the microwave material properties for freshly harvested seedcotton. Ideally this information could be obtained using high quality laboratory waveguide measurements. Unfortunately, there are several issues impeding this approach. The first is that loose seedcotton and cotton lint have very low densities and contains a significant amount of air in the sample. So much so that the permittivity of loose cotton lint is very close to that of air. Even in high-density cotton bales, the permittivity range is only from 2 to 2.5. Thus, the microwave permittivity of loose samples is much too low to provide useful results. To alleviate this, two approaches are possible: raise the frequency to 15–20 GHz or increase the density and the path-length of the waveguide. The first approach of increasing the frequency does not provide useful information, as the response will be significantly different than the targeted frequency range of interest, for reasons discussed in the earlier theory section. The second approach runs into the difficulty of trying to uniformly pack cotton to the target density without affecting the dimensions and microwave signal-integrity of the waveguide. Hence, after deliberating upon these options, the authors opted to perform the experiments directly on the samples of interest to the study as ultimately this is the measurement that the future sensor will need to perform. It was also felt that extrapolation from a small waveguide measurement to a full module could potentially provide a much greater source of error than the one obtained in extrapolating from a micro-module with a 0.61 m path length to a full-sized module with a 2.74 m path length.

The next challenge of importance, bearing in mind that the future prototype of interest is targeted for use on the harvester for real-time estimation of the moisture of seedcotton, is to ensure the material tested matches the material as presented to the sensor in its designed near real-time use. The most important of the issues relates to the fact that, as moisture migrates from surface-moisture to interior-moisture over time, these different types of molecular water attachment present significant differences in microwave responses. Therefore, to help minimize these effects, the test was designed to minimize the time between harvest and the experimental readings. Further, the experiments were designed such that the test cotton would span the normal operational ranges in density and moisture content, “MC”. This range in harvester-expected MC typically spans from 6% to 12% as seedcotton is only safe to store when it is at or below 12%. Thus, the critical zone for accuracy is to help determine the 12% threshold so the sensor can alert the operator that the crop is too wet for harvesting and that harvesting should cease. The objective of the research was to obtain a range of moisture contents and densities that are typical to freshly harvested seedcotton. The density range of interest for micro-modules and the new round modules was from 282 to 360 kg·m^−3^ (12.4 to 15.8 lbs·ft^−3^) (wet basis). The experiments were run according to commercial harvest practices utilizing a John Deere 7460 cotton stripper with the on-board field cleaner operating.

**Figure 4:**
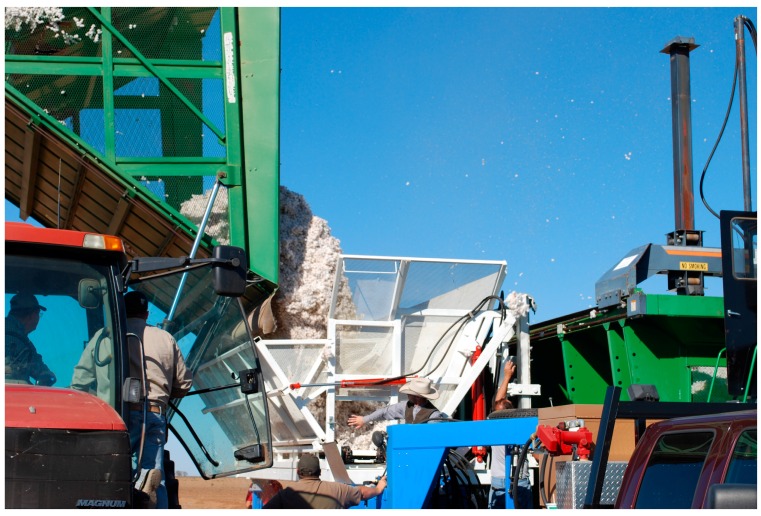
Harvester unloaded freshly harvested seedcotton into the instrumented weigh-cart/micro-module builder used to create the 110 kg micro-modules.

**Figure 5:**
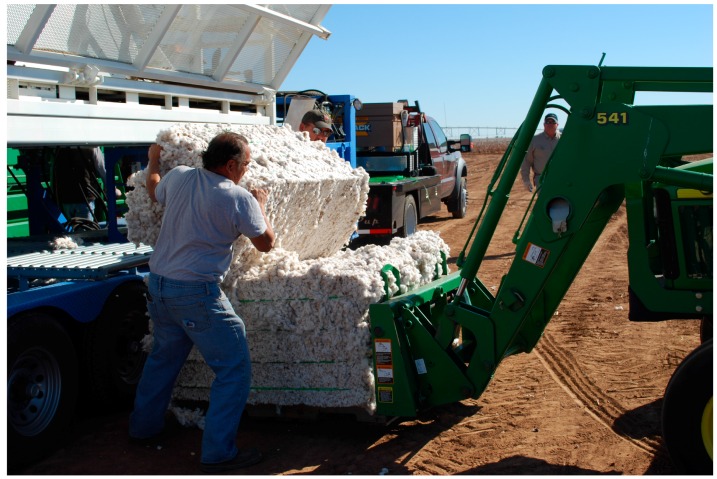
Micro-module exiting the weigh cart/micro-module builder.

The harvest operation was started early on a morning where the dew point temperature had dropped below saturation temperature. This ensured the seedcotton on the plants in the open bolls were subjected to at least 4 h of relative humidity, “RH” at 100% RH. The first micro-module was harvested early in the morning to obtain the first excessively wet module (22.4% MC); however, the cotton was too wet to push through the harvester and the machine clogged, so that only a partial module was obtained. After this initial module, the harvest operation was delayed for several hours to allow the moisture content to fall into a range where the cotton could be harvested. Once the cotton had dropped in to 12.5% MC, harvest was continuous with the objective to allow for the normal heating of the day to slowly adjust the seedcotton moisture, from wet to dry (7% MC), as the harvest operation progressed. To gain a variation in density as the modules were harvested, the amount of seedcotton added to each module was varied to obtain a range of module densities ranging from 282 to 360 kg·m^−3^ (12.4 to 15.8 lbs·ft^−3^). Given that free water behaves very differently than sorbed bound water, to ensure the microwave characterization matched what a real-time sensor on the harvester would encounter in use, the objective of the experiment was to perform the microwave characterization as close to the harvest time as possible. To achieve this, immediately after harvesting a single 240 m row; the harvester transported the freshly harvested seedcotton to an instrumented custom weigh cart that was configured with a micro-module press for building modules in-field. To help in the usage of the press; the cart was outfitted with a set of load-cells on each of the corners of the bulk bin that allowed for monitoring of the cart’s seedcotton weight. This scale was used to gauge how much seedcotton was removed from the bulk bin and conveyed into the micro-module builder (Figure 4) where it was pressed into a micro-module. During the transfer of the seedcotton from the cart’s hopper to its module builder, one sample was collected for fractionation analysis [30] and at least six samples were collected for moisture determination. The samples were immediately sealed into freezer bags for gravimetric analysis later that same day. After building each micro-module (Figure 5), they were immediately transported to the laboratory for microwave material characterization and weighing on a certified, legal for trade, cotton bale scale.

Of importance to microwave sensing of moisture with a small number of frequencies is phase ambiguity. This occurs when the measured relative phase delay exceeds 360°. Should this occur, the risk is that an excessively wet module could be mistaken by the sensor for a dry module. To guard against this potential, the experimental analysis also included the low-density excessively wet partial module in the dataset, as it represents a worst-case scenario for phase ambiguity. This bale turned out to be instructive as it provided unique insight into the extreme points in the trends between the various microwave properties, with respect to that of moisture and density. For each micro-module, the microwave response was measured on each module at three locations, one near both ends and one reading in the middle. The measurements were obtained with a Hewlett Packard 8753D microwave network analyzer (operational frequency 100 kHz to 6.0 GHz) utilizing a set of COM-POWER AH118 wideband double-ridge horn antennas (operational frequency 800 MHz to 20 GHz). The scan frequency was set to obtain microwave spectra from 800 MHz to 2.5 GHz, as 2.5 GHz was the expected upper end where the signal would not become too faint for a sensor to detect after passing through a full-sized module. At the beginning of the test, it was determined that above 2.5 GHz the attenuation on even the narrow path-length micro-modules was severe for the very wet module (in excess of −29 dB over micro-module’s 0.6 m path length). Thus, frequencies above 2.5 GHz were assessed to not be practical for the full-sized modules. To obtain the density; each module was weighed on a certified cotton bale scale, and the density was calculated by dividing by the known volume of the compression box that the module was built in (0.321 m^3^). The moisture content was measured with six samples taken from the interior of each module as it was being built, and then quantified by measuring the MC of each sample using standard oven gravimetric analysis for moisture determination [31], modified by accepted practice, USDA-ARS cotton ginning laboratories, of weighing the samples hot out of the oven on a precision balance.

## 3. Results

**Figure 6:**
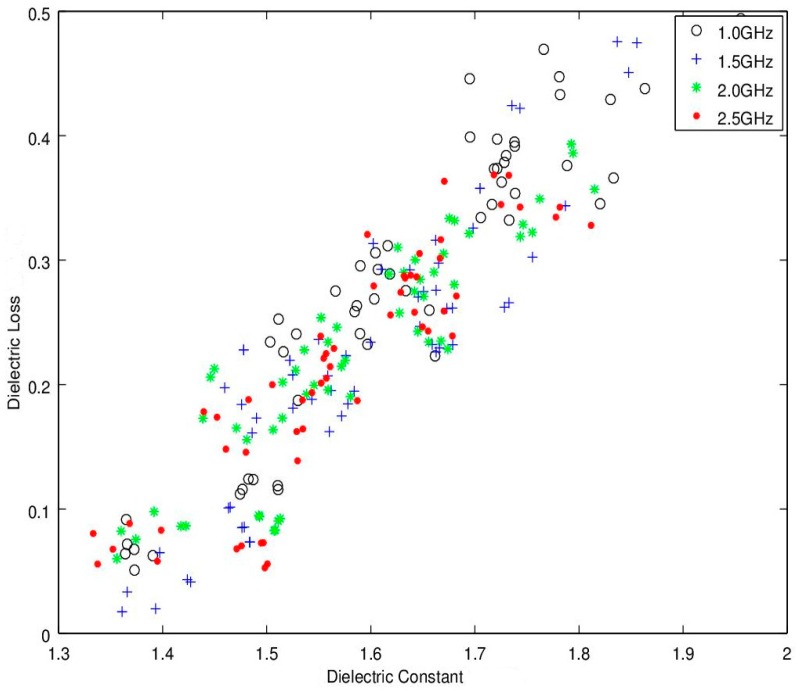
Picture illustrates the Cole-Cole response for several frequencies from 1.0 to 2.5 GHz (ε’ versus ε”). Over this narrow of a frequency range, only a small section of the Cole-Cole ellipse is represented.

**Figure 7:**
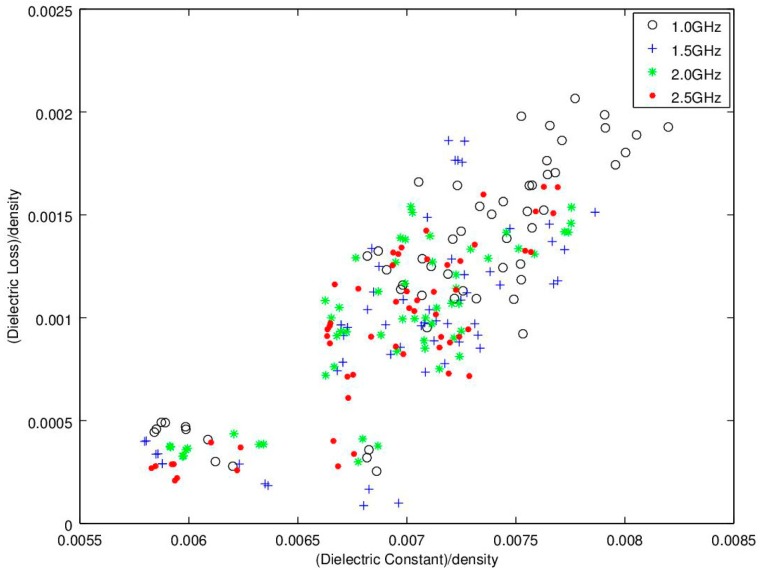
Detailed is a common approach showing the density-normalized Cole-Cole response as a function of frequency (ε’/ρ and ε”/ρ). Of interest is that previous researchers have reported this relation provides a linear output for agricultural seeds at higher microwave frequencies of 7, 11.3, 14.2, 18 GHz. Of importance is that, at this much lower frequency, the response shows a much higher degree of scatter and is therefore not likely to be useful for sensing moisture.

**Figure 8:**
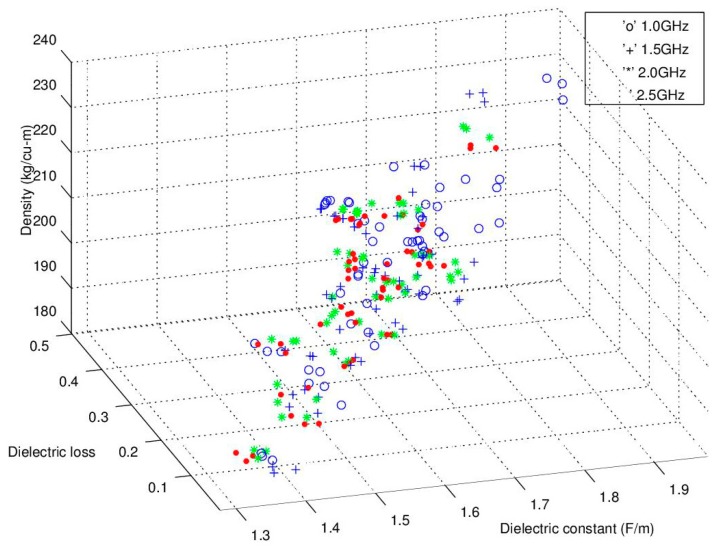
Cole-Cole plot of the response for the measured frequencies (1.0, 1.5, 2.0 and 2.5 GHz) with density shown on the *z*-axis.

The experiments were analyzed and the microwave spectral responses for each module were analyzed for assessment of the seedcotton’s microwave propagation properties. Noting that use of low-loss Equations (3) and (4) require that ε”/ε’ << 1.0. For the modules harvested in this test, the highest loss was found to occur in the wettest module at ε”/ε’ = 0.25. Thus, the experimental data confirmed the low-loss approximation equations can be utilized with minimal error. The next step for assessing the microwave properties was provided in the microwave sensing literature where it was reported that a linear relation occurs between ε’/ρ versus ε”/ρ with the notable feature that an increase along the *x*-axis (ε’/ρ was reported to also correlate well to an increase in moisture content. This relationship is in essence a density-normalized Cole-Cole response correlation (also known as an Argand-Diagram; [29]). For adoption of this relationship to the task of moisture sensing at the lower microwave frequencies of interest (1.0, 2.0 and 2.5 GHz), the Cole-Cole chart is illustrated in Figure 6. Significantly, this linear trend provides the primary basis for many of the reported density-independent functions as reported in; [16,23,28,29,32]. Unfortunately, it appears that at the lower frequencies the relationship predicted from the higher GHz region appears only modestly linear and lacks the finesse required for accurate sensor development. To gain further insight, the traditional Cole-Cole diagram is presented in Figure 7 and again in 3D with density on the *z*-axis in Figure 8. Reflection on these charts suggests that while this relation may work well above 8–10 GHz, it does not perform very well at the lower frequencies of interest to this research. It is surmised that potential reasons for the discrepancies are due to the much different dielectric response, and dielectric loss, of water in the low GHz region (Figure 3). There is also a portion of the deviation to be attributed to the significant deviation in density range, as the tests reported in the literature were over a much narrower density range of 0.72–0.85 g·cm^−3^ (wheat) and 0.69–0.83 g·cm^−3^ (corn), where the density change was basically limited to variations in the packing density of the seed kernels. In comparison, the density range of seedcotton spans a significantly larger span, ranging from 0.28–0.36 g·cm^−3^ (seedcotton) (40% greater coverage). The final point to note is that seedcotton constituents are comprised of only 30% seeds, the rest being predominantly cellulose, hemi-cellulose and lignin, which has a very complex relation to moisture and almost certainly has a much different specific surface area in comparison to the seed kernels of corn and wheat cereal grains.

In examining Figure 6, Figure 7 and Figure 8, of primary interest to this research is that the high scatter of the trend is so significant that it casts doubt that the basic predictive equations [20,21,22,23,25,26,27,28,29,32] will hold for use as a prediction basis for moisture content in seedcotton at these low microwave frequencies. This assessment will be explored in more detail in part two of this series.

Given the high scattering, utilizing the traditional density-normalized Cole-Cole approach, (ε’/ρ versus ε”/ρ) (Figure 6, Figure 7 and Figure 8), the search for additional relations were explored. The basic data for these alternative microwave parameters will be reported herein and the potential for their use in density-independent prediction equations will be examined in part two of this series.

**Figure 9:**
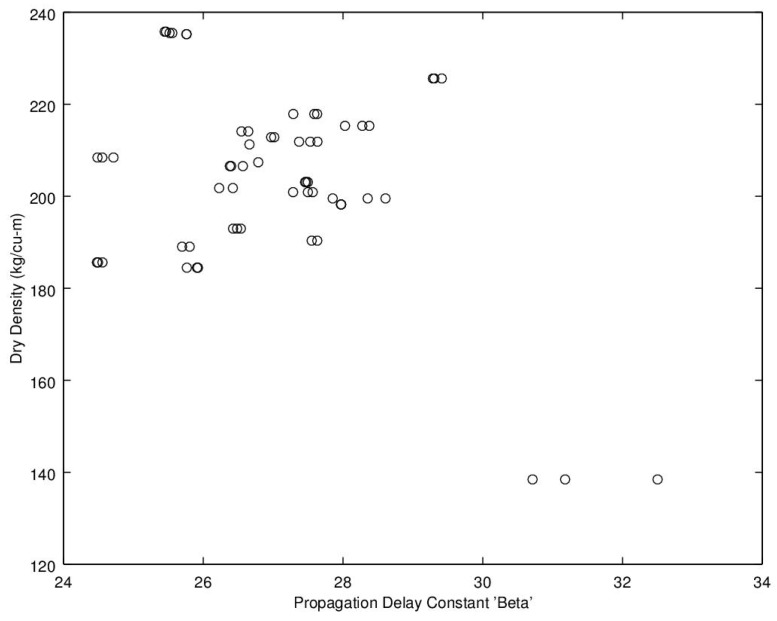
Propagation delay constant, β, versus bulk dry density (no water) at 1.0 GHz.

**Figure 10:**
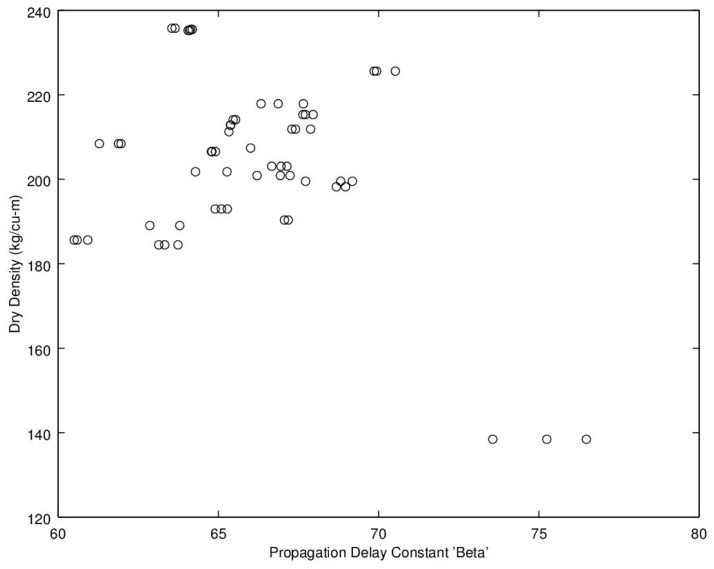
Propagation delay constant, β, versus bulk dry density (no water) at 2.5 GHz.

**Figure 11:**
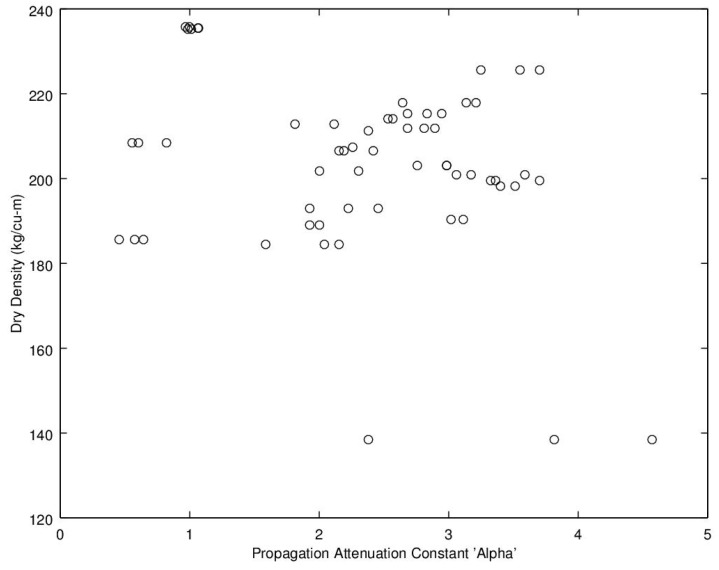
Propagation attenuation constant, α, versus bulk dry density (no water) at 1.0 GHz.

**Figure 12:**
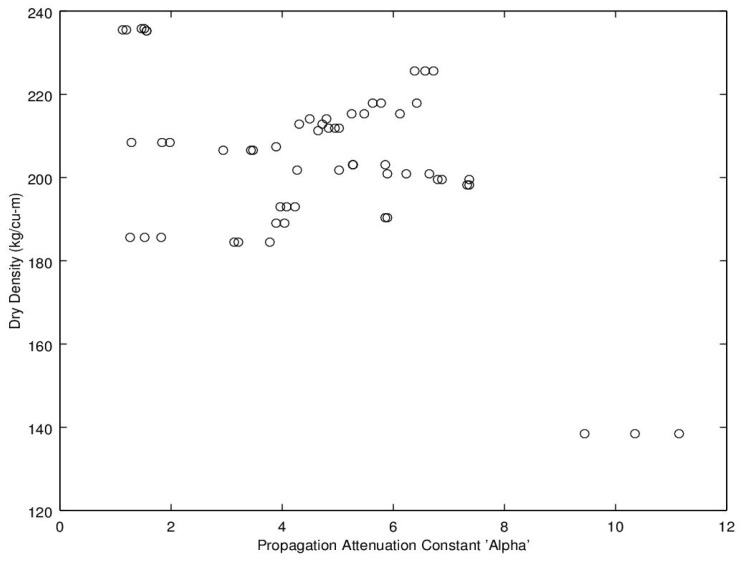
Propagation attenuation constant, α, versus bulk dry density (no water) at 2.5 GHz.

Noting the enhanced value of a sensor that does not require a weighing operation, of potential interest is to find a relationship between dry density (no water) and microwave propagation constants {β,α} (Figure 9, Figure 10, Figure 11 and Figure 12). Unfortunately, Figure 9 and Figure 10 show very poor correlation between the propagation delay term β and bulk dry density at 1.0 and 2.5 GHz. Similarly, poor results are detailed by Figure 11 and Figure 12 for the propagation attenuation constant α with respect to dry bulk density. This is significant as it proves that neither attenuation nor the orthogonal component of propagation constant, the delay term β correlate to the dry matter in the sample. Hence, it is conclusive that any correlation to β or α is predominantly due to the water in the seedcotton. That leaves, however, the question: Is there some minor variation in the response due to water being at a higher or lower bound water level that would provide some multi-variate equation to allow for prediction of MC independently of density? The observed experimental results suggest that is the most likely path to success. It also suggests that the dry bulk material is likely to only add noise to the dataset and should most likely be excluded from any multi-variate regression terms.

**Figure 13:**
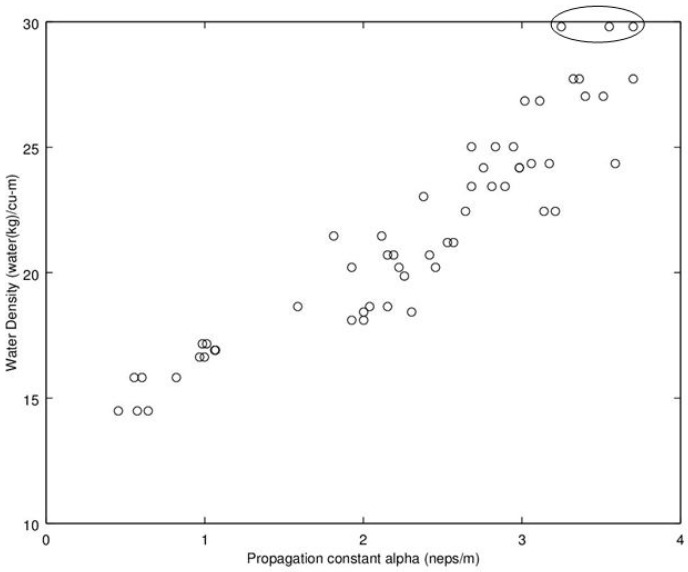
Graph detailing propagation attenuation constant, α, versus bulk water density at 1.0 GHz. Of note is the lack of predictive ability for the very wet low-density module. The points associated with the excessively wet low-density module are circled. Note water density calculated from moisture content utilizing Equation (9).

**Figure 14:**
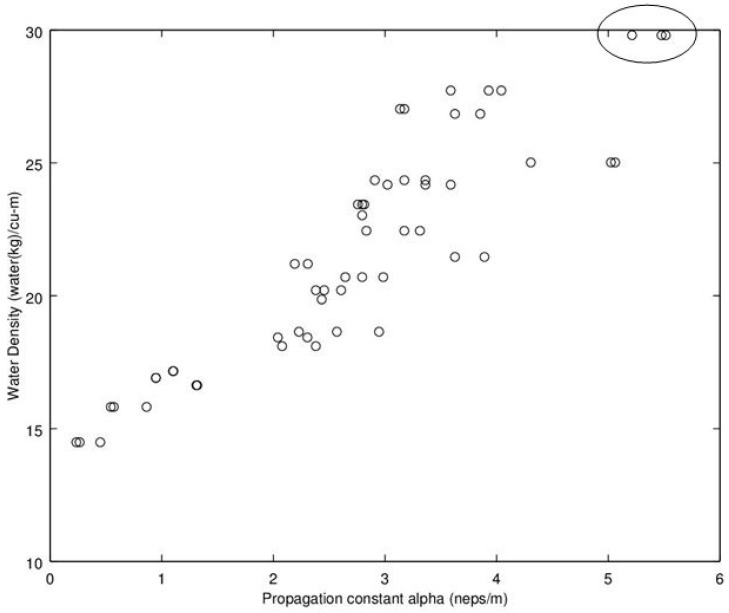
Graph detailing propagation attenuation constant, α, versus bulk water density at 1.5 GHz. Of interest is that the points on the excessively wet low-density module, circled, are moving closer together as the frequency increases. Note water density calculated from moisture content utilizing Equation (9).

**Figure 15:**
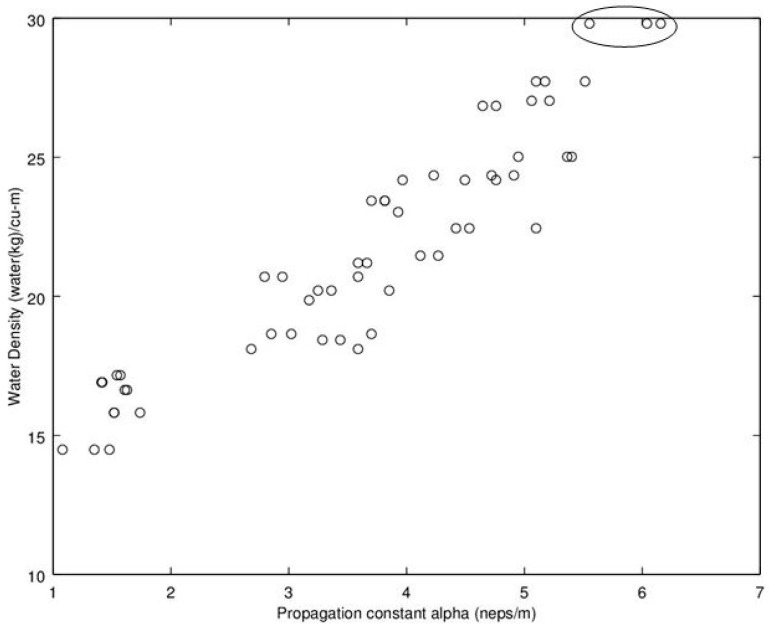
Graph detailing propagation attenuation constant, α versus bulk water density at 2.0 GHz. Of particular note is that at 2.0 GHz all the readings from the excessively wet low-density module are co-aligned (when they were not at the lower frequencies). The points associated with the excessively wet low-density module are circled. Note water density calculated from moisture content utilizing Equation (9).

**Figure 16:**
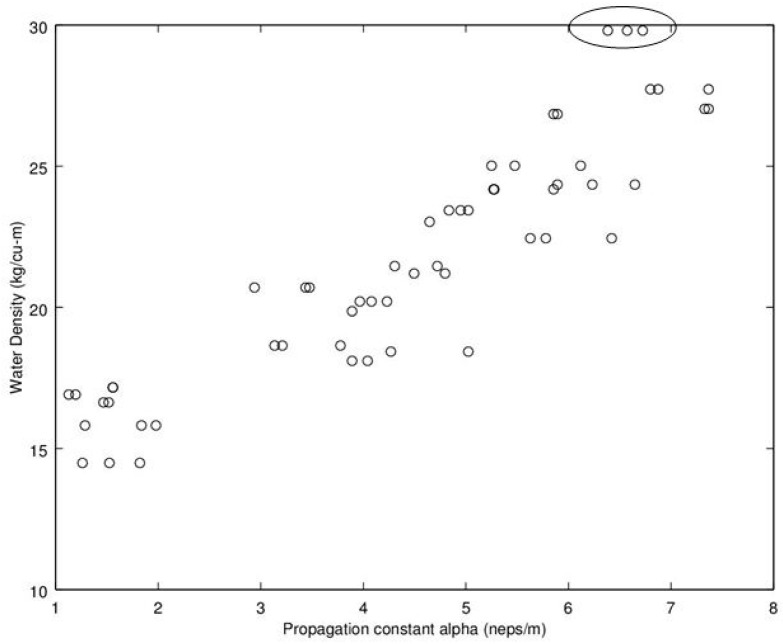
Graph detailing propagation attenuation constant, α, versus bulk water density at 2.5 GHz. Of interest is that as the frequency is moving still higher; the very wet low-density module samples are inverted and are again moving apart. The points associated with the excessively wet low-density module are circled. Note water density calculated from moisture content utilizing Equation (9).

The next aspect to examine was the correlation between the microwave parameters to MC, per the standard practice as reported in the literature. Given the lack of correlation and likely noise source attributed to the dry bulk density, the approach taken here deviates from the traditional approach, of relating microwave properties to MC. Instead, the approach substitutes water density, WD, (kg_water·m^−3^) (similar to volumetric water content) which was then found to exhibit a much higher correlation than that MC provides to microwave properties of {β,α} Given these positive results, Figure 13, Figure 14, Figure 15 and Figure 16 detail the correlation between the propagation attenuation constant α and bulk water density, at 1.0, 1.5, 2.0 and 2.5 GHz. Of note is that the results were obtained for micro-modules with a 0.61 m path length. The results of particular interest are the scatter in the excessively wet low-density module’s readings that are minimized at 2.0 GHz, which then expand back out again as the frequency increases to 2.5 GHz. As they have converged at 2.0 GHz, this suggests that the optimum frequency for using α in a prediction equation is at or near 2.0 GHz. This is a feature that should be considered for further exploration in future work to see if the trend holds across a more robust dataset than a single unique module.

**Figure 17:**
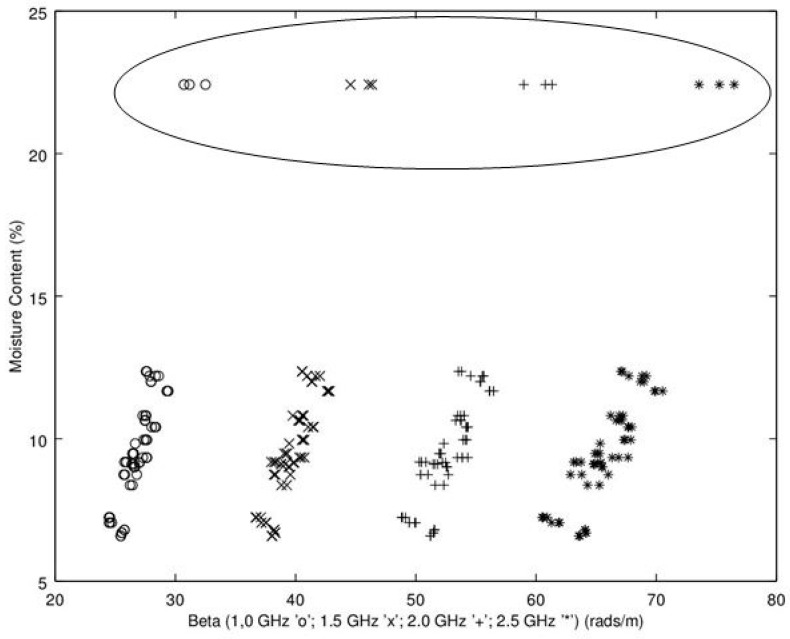
Propagation delay constant, β, versus moisture content at 1.0, 1.5, 2.0 and 2.5 GHz. The points circled are from the excessively wet low-density module.

**Figure 18:**
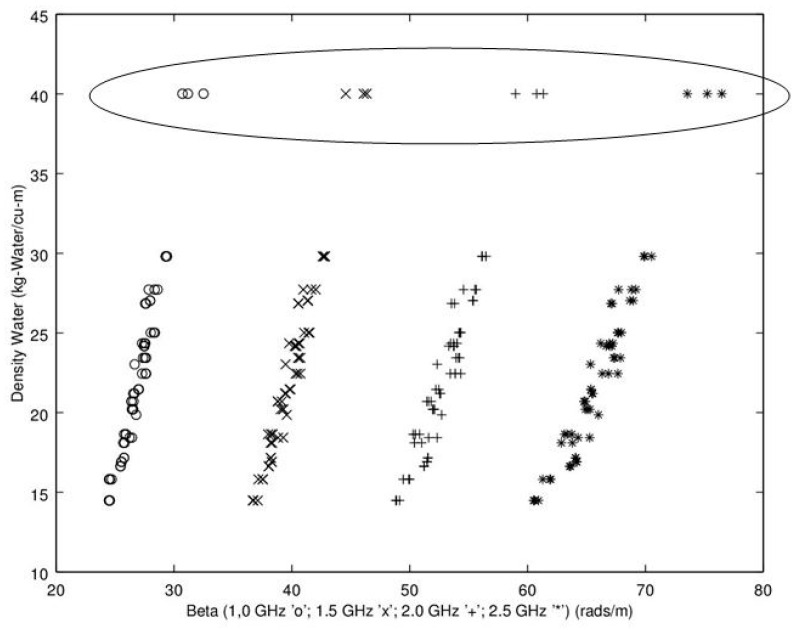
Propagation delay constant, β, versus bulk water density (kg_water·m^−3^) at 1.0, 1.5, 2.0 and 2.5 GHz. Of note is the much higher correlation than when β is compared to moisture content, thereby further illustrating that the microwave energy is almost entirely impacted by the water alone, not the dry bulk material. The points circled are from the very wet low-density module. Note water density calculated from moisture content utilizing Equation 9.

While the results provided by attenuation constant have much higher correlation to WD than dry bulk density, the correlations are too low to provide strong predictive ability. The complimentary candidate for microwave properties is the delay propagation constant β. For comparison to assess performance for use of WD versus MC, the phase delay β is shown in comparison to MC in Figure 17 and WD in Figure 18. The results for regression of β versus MC provides a much better relationship than α provided. There is however, still some curvature and a fairly extensive amount of scatter and correspondingly low r^2^ = 0.78 with a cross-validation root-mean-squared error, RMS_error_ = 1.98 (Table 1 details results; linear regression MC = f(β)). The most likely candidate for the high scatter is due to the wide variation in density, wet basis, in the dataset. To test this hypothesis, density is brought in as a co-variate, with improved results of r^2^ = 0.994 with a cross-validation RMS_error_ = 0.96 (Table 2; simple linear regression MC = f(β,ρ)).

Next, examination of the correlation to water density is performed (Figure 18). This figure provides further evidence that microwave properties are due almost solely to the water in the material. Correlation analysis produced a coefficient of determination of r^2^ = 0.950. When tested as a co-variate, module density was not found to be significant and did not improve the regression. The results suggest that any of the frequencies could be utilized in a prediction equation to estimate WD. To transform from WD to MC, the simple transformation equation is shown in Equation (9). MC = Water Weight/ (Water Weight + dryWeight)

MC = WD (kg_water·m^−3^)/[Wet_Density((kg_water + kg_dryMatter) m^−3^)] (9)

where

Wet_Density ≡ (wet weight of the module)/(volume [m^3^])
WD ≡ Water_Density ≡ (weight of water)/(volume [m^3^])

To test the performance of this new method of obtaining moisture content, from a prediction of WD, the MC was calculated from the WD prediction utilizing the known weight of the module, per Equation (9). To ensure accurate assessment of predictive accuracy, all results reported are cross-validated with the coefficient of determinations reported as adjusted r^2^. The accuracy of predicting the MC using this approach produced, for a mono-frequency second-order linear regression (MC = f(β,β^2^) [1.5 GHz]), an adjusted r^2^ = 0.963 with an rms_error_ = 0.63% MC, which was a modest improvement over the direct prediction of MC using density and a second-order function of β.

## 4. Conclusions

The rapid and reliable assessment of moisture content on cotton harvesters and for in-field data collection is a valuable tool needed for agronomic field trials. The results reported herein provide the basic microwave material properties required to develop a prototype sensor for measuring the moisture content stripper-harvested seedcotton packaged in bulk micro and commercial modules. The results indicate that for large bulk packaged seedcotton, the use of lower microwave frequencies will be required because, at higher frequencies, the signal loss is too great. This was found as the attenuation for 2.0 GHz and 2.5 GHz was over 25 dB of loss for the wettest micro-modules. Given the path length tested was only 0.61 m, if a similar system was utilized for commercial scale modules with a 2.74 m path length, the extrapolated loss would be >80 dB. For micro-modules, any of the frequencies reported herein could be utilized. For commercial modules, the use of the lowest bands tested herein is recommended, as a very wet commercial-sized module would likely be undetectable (which could also be a technique to flag a very wet module). However, care should be exercised as multi-path signals could very well work their way around the module and then provide a false “dry” bale measurement. Hence, this method is not recommended. It is possible that even lower bands below 1 GHz may prove useful as well. For use in the US, the test results presented herein suggest that the license-free 915 MHz scientific band is expected to produce the best balance between accuracy, reliability for prediction of seedcotton module moisture. For other countries such as Australia and EU member states, a survey of legal scientific bands, with respect to the results published herein, is recommended and left to the reader.

A key finding revealed by this study is that the literature-reported X-band (10 GHz) density-independent moisture content (MC) prediction equations do not perform the same at the lower frequencies (1–2.5 GHz) necessary for seedcotton. The cause of the deviations between the literature 10+ GHz prediction equations and the results presented herein are left for future work. It is surmised that they are likely due to variation in internal molecular and physical structures between seedcotton constituents, versus corn and wheat. An alternative explanation is that it could simply be due to the water’s different response at these lower microwave frequencies. The test results also determined that none of the commonly used microwave parameters, explored herein or published in the literature, have predictive ability for measuring the dry bulk density of seedcotton.

For moisture content assessment, without a known weight on the module, using a mono-frequency measurement of β (at 1.0 GHz) produced an adjusted r^2^ = 0.781 with a cross-validation RMS_error_ = 1.98% MC. In comparison; the accuracy of predicting the MC using the alternative approach to first predict water density and then utilize the known module weight to compute MC produced the best results with an adjusted r^2^ = 0.963 with a cross-validation RMS_error_ = 0.63% MC. The cross-validation accuracy over the reduced span of the normal expected operating range improves the results to an RMS_error_ = 0.48% MC with an adjusted r^2^ = 0.921. Part two of this series will examine in more detail the potential usage of density-independent moisture content prediction equations.

## Figures and Tables

**Table 1:** Results for prediction of moisture content using β, phase delay (density unknown).

Frequency (GHz)	RMS Error	r^2^
1	1.98	0.781
1.5	2.12	0.797
2	2.2	0.781
2.5	2.2	0.782

**Table 2:** Results for prediction of moisture content using β, phase delay (density included as co-variate).

Frequency (GHz)	RMS Error	r^2^
1	0.96	0.914
1.5	1.07	0.893
2	1.08	0.892
2.5	1.1	0.888
